# The Influence of Tea Tree Oil on Antifungal Activity and Pharmaceutical Characteristics of Pluronic^®^ F-127 Gel Formulations with Ketoconazole

**DOI:** 10.3390/ijms222111326

**Published:** 2021-10-20

**Authors:** Magdalena Wróblewska, Emilia Szymańska, Katarzyna Winnicka

**Affiliations:** Department of Pharmaceutical Technology, Faculty of Pharmacy, Medical University of Białystok, A. Mickiewicza 2c, 15-222 Białystok, Poland; esz@umb.edu.pl

**Keywords:** tea tree oil, ketoconazole, antifungal activity, Pluronic^®^ F-127, gels, in vitro release, permeation

## Abstract

Fungal skin infections are currently a major clinical problem due to their increased occurrence and drug resistance. The treatment of fungal skin infections is based on monotherapy or polytherapy using the synergy of the therapeutic substances. Tea tree oil (TTO) may be a valuable addition to the traditional antifungal drugs due to its antifungal and anti-inflammatory activity. Ketoconazole (KTZ) is an imidazole antifungal agent commonly used as a treatment for dermatological fungal infections. The use of hydrogels and organogel-based formulations has been increasing for the past few years, due to the easy method of preparation and long-term stability of the product. Therefore, the purpose of this study was to design and characterize different types of Pluronic^®^ F-127 gel formulations containing KTZ and TTO as local delivery systems that can be applied in cases of skin fungal infections. The influence of TTO addition on the textural, rheological, and bioadhesive properties of the designed formulations was examined. Moreover, the in vitro release of KTZ, its permeation through artificial skin, and antifungal activity by the agar diffusion method were performed. It was found that obtained gel formulations were non-Newtonian systems, showing a shear-thinning behaviour and thixotropic properties with adequate textural features such as hardness, compressibility, and adhesiveness. Furthermore, the designed preparations with TTO were characterized by beneficial bioadhesive properties. The presence of TTO improved the penetration and retention of KTZ through the artificial skin membrane and this effect was particularly visible in hydrogel formulation. The developed gels containing TTO can be considered as favourable formulations in terms of drug release and antifungal activity.

## 1. Introduction

Topical drug delivery is recognized as an effective method of therapy, which is a desirable feature for the relief of local symptoms at a relatively low dose, hence reducing systemic side effects. Pluronic^®^ F-127 (amphiphilic copolymers consisting of units of ethylene oxide and polypropylene oxide) gels have been widely investigated in the literature as drug carriers because of their low toxicity, reverse thermal gelation, high drug loading capabilities, and ability to gel in the physiological conditions at relatively low polymer concentration [1,2,3]. Pluronic lecithin organogel (PLO) is an interesting vehicle owing to its biocompatibility, amphiphilic nature, facilitation of drug dissolution, and its permeation enhancement properties. It is a two-phase system consisting of the oil phase and the water phase. PLOs are nonirritant two-phase formulations, composed of phospholipid (lecithin) as surfactant, an organic solvent as the outer continuous phase, and a polar water phase. The entangled reverse micelles form a three-dimensional network which entraps the outer continuous nonpolar phase and immobilizes it, turning into a viscous gel by self-association of separate gelator molecules [2,4,5]. Different physicochemical types of drugs can be easily incorporated into PLO gels. A number of studies have shown that PLOs have the unique capacity to deliver drugs through the skin [6,7,8,9,10,11,12,13].

Fungal infections are one of the most commonly encountered skin diseases. Skin mycoses affect up to 25% of the global population [14,15]. The primary treatment for superficial fungal infections is antifungal topical formulations [16,17,18]. Azoles represent one of the major classes of antifungal compounds which are used in topical formulations. Ketoconazole (KTZ), an imidazole derivative, is known as a broad-spectrum agent active against a wide variety of fungi and yeasts. KTZ is used both in the treatment of topical and systemic fungal infections. It interferes with the fungal synthesis of ergosterol, a constituent of cell membrane specific to fungi, and inhibits the biosynthesis of triglycerides, phospholipids, and oxidative or peroxidative enzyme activity, resulting in intracellular buildup of toxic concentrations of hydrogen peroxide. In the treatment of *Candida albicans* infections, KTZ also inhibits the transformation of blastospores to invasive mycelial forms [19]. KTZ is lipophilic and practically insoluble in water (0.0866–40.0 µg/mL; logP 4.31), which may influence its therapeutic efficiency [20,21].

Tea tree oil (TTO) is the essential oil obtained by steam distillation from *Melaleuca alternifolia* and it is used medicinally as a topical antiseptic. It has a broad spectrum of antimicrobial activity against a wide range of bacteria, viruses, and fungi, including yeasts and dermatophytes. TTO is a mixture of more than 100 different compounds, primarily terpenes, monoterpenes, and sesquiterpenes. The most important component in this oil because of its confirmed antimicrobial properties is terpinen-4-ol [22,23,24,25,26]. Due to the increase in infections that are resistant to antibiotics or chemotherapeutics, TTO may be used as an alternative therapy or in combination with conventional drugs to enhance their action [25,27]. While monotherapy or combined therapy based on conventional drugs is ineffective, a combined treatment including a natural agent might be more efficient. Several studies have reported the increased antimicrobial activity of natural substances combined with synthetic drugs as compared to conventional drug treatment alone [27,28,29,30].

The purpose of this study was to develop and characterize Pluronic^® ^F-127 gel formulations (hydrogels and pluronic lecithin organogels (PLOs) based on isopropyl myristate, castor oil, and paraffin oil) with TTO and KTZ intended for dermal application in the case of fungal infections. The physicochemical, mechanical, and bioadhesive properties of the prepared formulations were examined. Moreover, the release of KTZ in vitro from the prepared gel systems, the permeation of KTZ through the artificial skin membrane, and its antifungal activity against the selected *Candida* strains were examined.

## 2. Results and Discussion

### 2.1. Solubility Studies of KTZ

Solubility is one of the essential parameters to achieve desired drug concentration at the site of application for optimal pharmacological effect. Low aqueous solubility is the major problem encountered with formulation development [31,32]. The active compound to be absorbed must be present in the form of solution, so proper selection of the solubility enhancement method is key to ensuring the goals of a good formulation, like high bioavailability, reduction frequency of dosing, and better patient compliance. The selection of the method for solubility enhancement depends upon drug characteristics like solubility, chemical nature, melting point, absorption site, physical nature, and pharmacokinetic behaviour. Various techniques are used for the enhancement of the solubility of poorly soluble drugs, which include physical and chemical modifications of the drug and other methods like particle size reduction, crystal engineering, salt formation, solid dispersion, and the use of co-solvents or surfactants [31,33]. In pharmaceutical technology, a popular method is the use of co-solvents, which affect the hydrophobic interactions and allow a system with an appropriate dielectric constant for the active substance to be obtained [31]. Developing a suitable vehicle for the topical administration of an active compound requires finding a balance between various elements, including solubility of the drug as well as the penetration enhancer content and the occlusive effect of the vehicle. The active compound solubility in the vehicle should be adequate to provide the full intended drug dose in solute form while ensuring the physical stability of the preparation at the same time [33,34].

KTZ is a lipophilic compound with low water solubility. It also has been shown to be unstable in aqueous solutions, which may result in reduced or lost activity as well as the formation of toxic degradation products [19,20]. Improving KTZ solubility may significantly affect the bioavailability and absorption from the application area, and thus lead to an increase in its therapeutic effectiveness.

In the first stage of the study, the solubilities of KTZ in water, isopropyl myristate, castor oil, and paraffin oil as well as the abovementioned solvents with the addition of 5% TTO were assessed. On the basis of the obtained results (Table 1), it can be concluded that the solubility of the KTZ was the lowest in water (9.56 ± 0.09). Moreover, the data also show that KTZ solubility was the highest in isopropyl myristate (93.99 ± 3.26) and castor oil (93.55 ± 8.39). The study also revealed that the addition of 5% TTO significantly increased the solubility of KTZ in all the tested solvents. TTO improved the solubility of KTZ in paraffin oil 5-fold, almost 4-fold in water, and about 2-fold in the case of isopropyl myristate or castor oil.

### 2.2. Preparation and Physicochemical Characteristics of the Prepared PLU^®^ F-127 Hydrogels and PLO Organogels

Preparations intended for use on the surface of skin affected by fungal infection should be characterized by high antimicrobial effectiveness, favourable application properties, and satisfactory release of the active substance from the vehicle. When developing semisolid drug forms, the choice of vehicle and excipients plays a crucial role, because they might affect the release of the active substance and the stability of the formulation. A suitable vehicle should be compatible with the active compound, ensure its chemical stability, and be characterized by good adhesion to the skin surface, which allows the drug to remain at the application site longer. Moreover, the vehicle should not cause allergic reactions and irritation.

Gel systems are promising carriers for the topical delivery of drugs. Pluronic^®^ F-127 is a non-toxic, biocompatible, and thermoactive copolymer capable of gelling at body temperature, so it can be used for the preparation of both hydrogels and organogels intended for local drug administration. Hydrogels based on this polymer are thermosensitive three-dimensional structures that ensure gelation of the preparation under physiological conditions. Moreover, they are characterized by low toxicity and favourable application properties [35,36]. Pluronic/lecithin organogels (PLOs) are amphiphilic systems that allow the dissolution of both lipophilic and hydrophilic active substances and can facilitate the drug penetration through the lipid layer of the epidermis. PLO gels are biocompatible and do not cause skin irritation [2]. As a part of this work, compositions of various gel system types based on Pluronic^®^ F-127 were developed. The hydrogel and three kinds of PLO organogels were prepared (Table 2). The formula of PLO gels included an oil phase containing phospholipid lecithin as a stabilizer and permeation enhancer; isopropyl myristate, castor oil, and paraffin oil as a solvent for lecithin and emollients; and an aqueous phase composed with Pluronic^®^ F-127 as a gelling agent, purified water as a solvent, and potassium sorbate as a preservative. Stable organogels were obtained with a water to oil ratio of 70:30. Hydrogels and PLO organogels were white or light yellow in colour and were characterized by uniform, smooth consistency, were free from grittiness, and did not show a tendency to syneresis. The prepared formulations were homogeneous and easily spreadable on the skin surface. Additionally, the preparations containing TTO were characterized by a spicy mint camphor scent.

To estimate the pharmaceutical and application properties of the designed gel formulations’ drug content, pH, KTZ particle size, and rheological and mechanical features were examined. Based on the analysis of the percentage of active substance content (Table 3), it was found that the KTZ amount in the prepared gel systems was in the range of 90–110%, which corresponds to the USP requirements for dermatological preparations with ketoconazole [37].

In the case of formulations containing only KTZ, the drug content was found to be in the range of 91.0–96.4%, while for preparations with KTZ and TTO it was 90.4–96.8%. The obtained values might indicate the uniform distribution of the drug in the vehicle and the lack of active substance degradation. For the development of semisolid drug forms intended to be applied to the skin, it is necessary to ensure the appropriate pH, which affects the stability of the active substance and the safety of the product to eliminate the risk of irritation. The pH value of the preparations applied on the skin surface should be close to its physiological pH, which is in the range of 4–6 [38]. The pH of the product should be matched to ensure the stability of the active substance. According to the literature, KTZ shows the highest stability at pH = 6–8 [20]. The pH of all prepared formulations was in the range of 6.5–6.8 (Table 3) and it was only slightly higher than the pH of the commercially available preparation, which was 6.4. The highest values of the determined parameter were obtained for hydrogel systems, and the lowest were obtained for organogels based on paraffin oil. In the case of formulations with the addition of TTO, a small decrease in pH was observed. However, the pH values obtained during the experiment corresponded to the skin pH and were suited for topical application [38].

During the development of semisolid topical drug carriers, it is important to ensure the proper size of particles suspended in the vehicle. The size of the dispersed particles is important for application reasons. Particles which are too large may be perceptible during application, causing unpleasant graininess or even inducing skin irritation. Furthermore, it may affect the release of the drug from the dosage form and the penetration through the skin, remarkably influencing the effectiveness of the preparation. The active substance should be uniformly distributed in the vehicle, and the particles should be of a suitable size, because smaller particles dissolve faster, providing better absorption. From the data presented in Table 3, it can be seen that the particles size in all the prepared formulations did not exceed 90 µm, the limit required in the case of topical formulations [39]. In formulations containing only KTZ, the average particle size was in the range of 58–63 µm, while in gels with KTZ and TTO, the particles were smaller and their size ranged from 36 to 55 μm, what may be the result of KTZ’s better solubility in the presence of TTO (Table 1).

**Figure 1:**
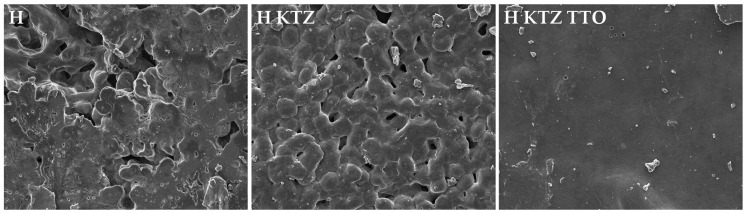
The SEM images of PLU^®^ F-127 hydrogels: placebo (H); with KTZ (H KTZ); and with KTZ and TTO (H KTZ TTO), magnification 1000×.

To investigate the impact of TTO on gel surface morphology, in the case of PLU^®^ F-127 hydrogels, as formulations characterized by the best antifungal activity and KTZ release, SEM experiments were performed. It was noted that hydrogel with the addition of TTO (H KTZ TTO) possessed dense, uniform, and regular structure (Figure 1). Due to introducing TTO into the formulation, the internal conformation of hydrogel appeared visibly more compact and less porous.

The rheological features affect the quality of the preparation, the drug release, and the application properties. Formulations intended for administration on the skin surface should have an appropriate viscosity, which determines the flow property, good spreadability, and extends the time the preparation remains at the application site, resulting in better patient compliance. Higher viscosity values prevent the formulation from draining off the skin surface. In addition, the higher viscosity in the case of the suspension type drug prevents sedimentation of the active substance particles and can ensure a homogeneous consistency of the product despite the longer storage time. Viscosity is also related to the mechanical properties and may influence the release of the active ingredient from the vehicle. It was found that, as the viscosity of the formulation increases, the amount of the released active substance decreases [11,40,41]. The conducted analysis shows that the prepared gel systems were characterized by significant differences in viscosity (Table 3). The prepared gels possessed viscosity values in the range 2128–23,548 mPa·s. Formulations containing KTZ were characterized by higher viscosity values compared to the placebo formulations. Moreover, it was noticed that the addition of TTO significantly increased the viscosity of all the analyzed preparations. This might be due to the formation of a complex network-like structure in the gels. A growth in viscosity was particularly visible in the case of the hydrogel, where an 11-fold increase in the analyzed parameter was observed. TTO in a concentration of 5% caused a 1.5-fold increase in the viscosity of the organogel with isopropyl myristate and a 2-fold increase in the examined parameter for PLO based on paraffin oil. In the case of PLO based on castor oil, there was a 3-fold increase in the viscosity after the addition of TTO. For all gel formulations, the recorded viscosity values ranged in the specific field suitable for semisolid preparations [40,41]. In the work of Mut et al., it was observed that the presence of essential oil in the formulation affected the viscosity of the study’s hydrogels. Cinnamon oil in a concentration of 3% increased the viscosity of the control gel by 1.32 times [42]. The differences in viscosity may be attributed to the changes in the polymer gels’ three-dimensional structure, because of different polarities and multiple chemical compositions of the TTO, as well as to possible interactions between the molecules of different components such as water, solvents, terpenes, and macromolecular ingredients [42]. The more compact structure of the hydrogel with the addition of TTO was visible in the SEM images (Figure 1).

**Figure 2:**
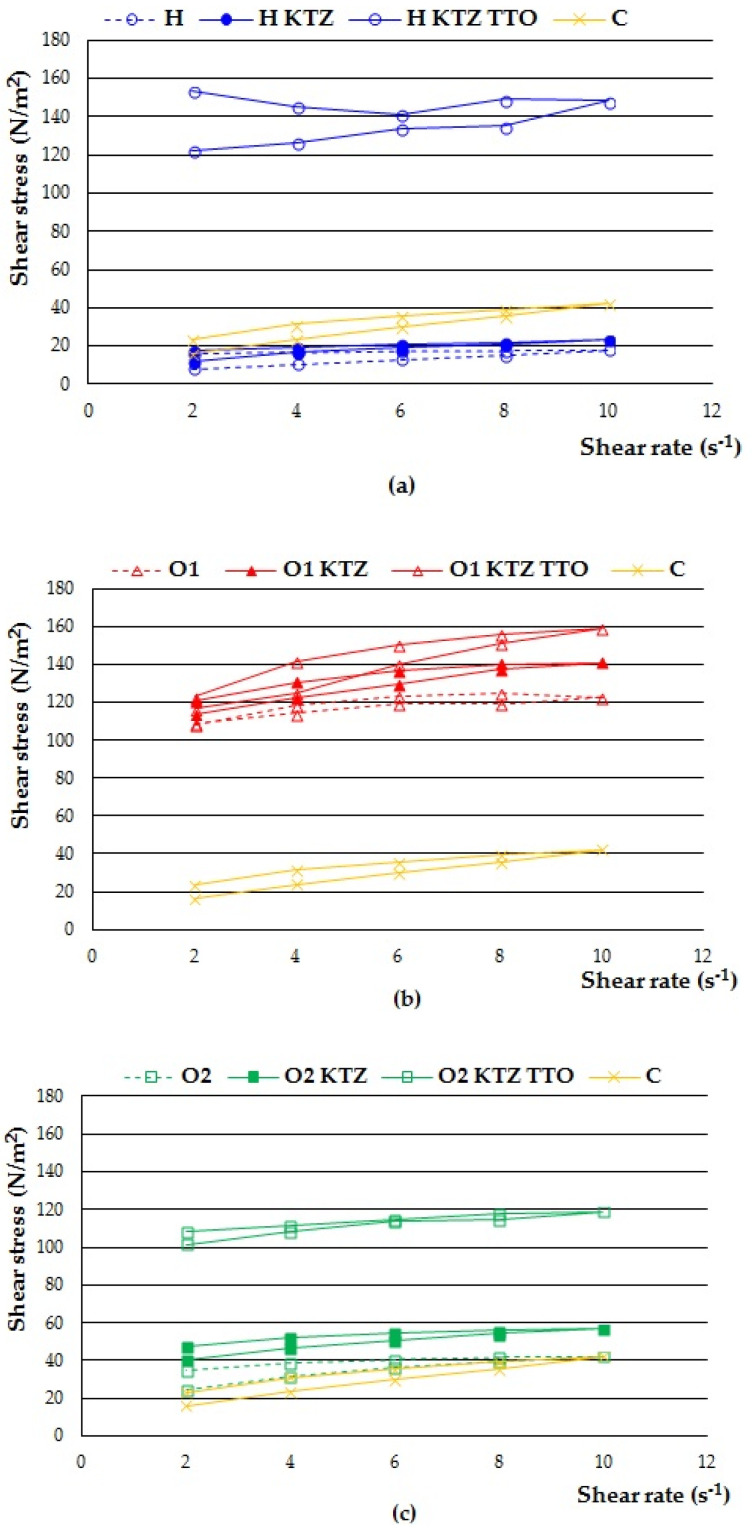

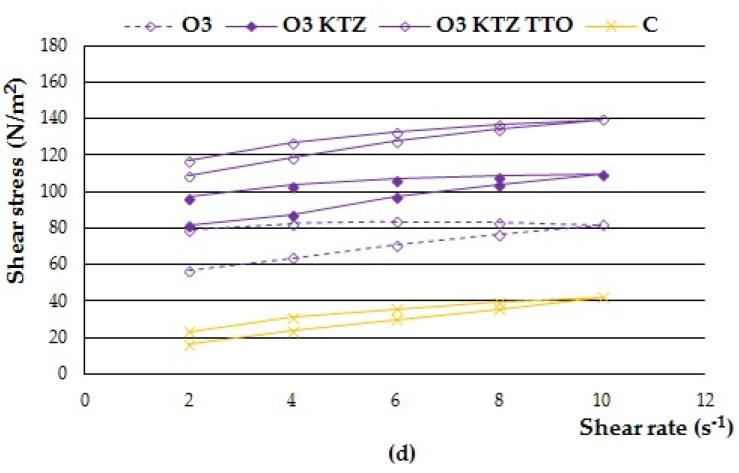
Rheograms of prepared PLU^®^ F-127 hydrogels: (**a**) H, H KTZ, H KTZ TTO, and PLO organogels; (**b**) O1, O1 KTZ, and O1 KTZ TTO; (**c**) O2, O2 KTZ, and O2 KTZ TTO; and (**d**) O3, O3 KTZ, O3 KTZ TTO, and commercially available product (C).

On the basis of the conducted research, it was also shown that the prepared formulations behaved like non-Newtonian, shear thinning systems. The viscosity of the gels decreased with the increase of the shear rate, which is a typical property of semisolid formulations. The shear-thinning behaviour is considered as very beneficial to the topical application of formulations, since this ensures the formation of a thin layer of the gel spread over the skin surface, resulting in a more efficient delivery of bioactive agents. The hysteresis loops presented in Figure 2 allowed the tested gels to be classified as thixotropic systems. They are characterized by their ability to rebuild the three-dimensional structure after shear stress, which previously led to the breaking of bonds between polymer molecules. The shear thinning flow and thixotropic behaviour are preferred for pharmaceutical dermal formulations to facilitate preparation, spreading, handling, and application to the skin. The obtained results are in accordance with other publications describing PLOs as topical drug carriers [2,5,7,10,11].

During the development of pharmaceutical semisolid preparations for topical application, several desirable product characteristics, such as hardness, cohesiveness, and adhesiveness, may be defined with the use of mechanical properties. Hardness is described as the force required to attain a given deformation. Compressibility is defined as the force per unit time required to deform the product during the first compression cycle of the probe. Adhesiveness is a quantity that simulates the work required to overcome the attractive forces between the surface of the sample and the surface of the probe with which the sample comes into contact. These parameters ensure easy application of the vehicle and removal from the packaging container, spreadability of the preparation on the skin, and good adhesion to the skin surface. Gel hardness, which expresses the applicability of the gels to the skin, or adhesiveness, which can be an indicator for the retention time on the site of administration, are directly correlated to the properties of the drug vehicle. Semisolid preparations employed for application to the skin surface, particularly in respect to prolonged retention time at the site of administration, should be characterized by high adhesiveness to ensure a longer contact time of the preparation at the site of application [41,43].

The study showed that the prepared placebo gel systems showed lower hardness, cohesiveness, and adhesiveness compared to gels containing KTZ or KTZ and TTO (Table 4). The addition of KTZ to all the prepared formulations caused a slight growth in the tested parameters, while loading 5% TTO significantly increased the values of hardness, cohesiveness, and adhesiveness. It was observed that the mechanical properties of all the gel preparations were affected by the addition of TTO. It may be concluded that the addition of TTO increases the network strength, and the mechanical properties are governed by the arrangement of the network.

### 2.3. Ex Vivo Bioadhesive Properties

Bioadhesion is defined as the interaction between biological materials or between biological material and a synthetic surface, which leads to their joining due to the production of interfacial energy. This process allows for the estimation of the ability of the preparation to stay at the site of application and to determine its adhesion strength to the surface. Due to its bioadhesive properties, it is possible to extend the action of the drug by increasing the time of its contact with the skin. The presence of polymers in the composition of the prepared gel systems ensures their good adherence to the skin, and thus a longer time at the application site [44].

**Figure 3:**
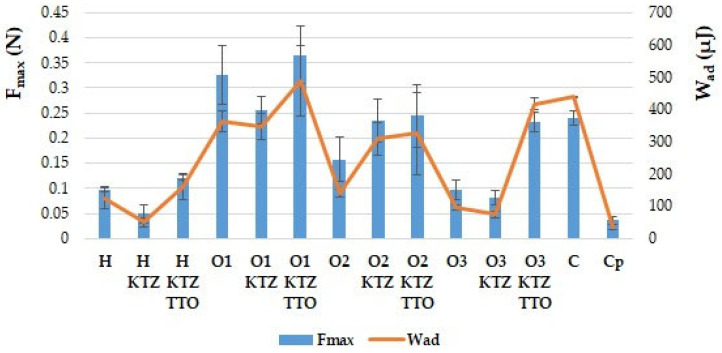
Ex vivo bioadhesive properties of prepared PLU^®^ F-127 hydrogels and PLO organogels containing KTZ (H KTZ, O1 KTZ, O2 KTZ, O3 KTZ), KTZ and TTO (H KTZ TTO, O1 KTZ TTO, O2 KTZ TTO, O3 KTZ TTO), placebo gels (H, O1–O3), and controls: commercially available product (C) and cellulose paper (C_p_) determined as the maximum detachment force (F_max_) and the work of adhesion (W_ad_). Values are expressed as means ± SD of six replicates; *p* ˂ 0.05.

The data presented in Figure 3 show that all prepared gel formulations, tested ex vivo with the use the of hairless mice skin as a model of the adhesive layer, were characterized by bioadhesive properties.

The placebo formulations possessed slightly higher F_max_ and W_ad_ values compared to the formulations containing the KTZ, with the exception of PLO based on castor oil, where greater values of the analyzed parameters for placebo formulation were noted. Furthermore, the addition of TTO led to an increase in both the tested parameters for all the designed gel formulations. The highest values of F_max_ and W_ad_ were observed in the case of the organogel based on the isopropyl myristate placebo, as well as this organogel containing KTZ and TTO, which were also characterized by high mechanical parameters and the highest viscosity (Table 2 and Table 3). It is assumed that preparations characterized by high viscosity and superior values of F_max_ and W_ad_ better adhere to the skin, and thus remain at the application site for longer [40]. On the other hand, the lowest values of F_max_ and W_ad_ were recorded for hydrogel preparations, as well as for PLO based on paraffin oil containing KTZ and placebo.

### 2.4. In Vitro Release of KTZ

The release of a drug from delivery systems depends on many physical and chemical properties of both the drug and the carrier, such as their chemical composition, molecular weight, degradation rate, particle size, and drug-matrix interaction. Testing the release of the active substance in the in vitro conditions enables the assessment of qualitative changes in the composition of the formulation, determination of the stability of the active substance in the preparation, and detection of potential interactions between the carrier and drug. The release of an active ingredient from dermatological preparations is significantly influenced by the partition coefficient between the vehicle and water, the solubility of the active substance in the acceptor medium and the vehicle, and the type of carrier and its viscosity. It is assumed that the release rate of the active compounds from hydrophobic bases or those in the form of emulsions is lower than that from hydrophilic vehicles and can be classified as follows: lipophilic ointment < O/W cream < hydrogel [45,46].

**Figure 4:**
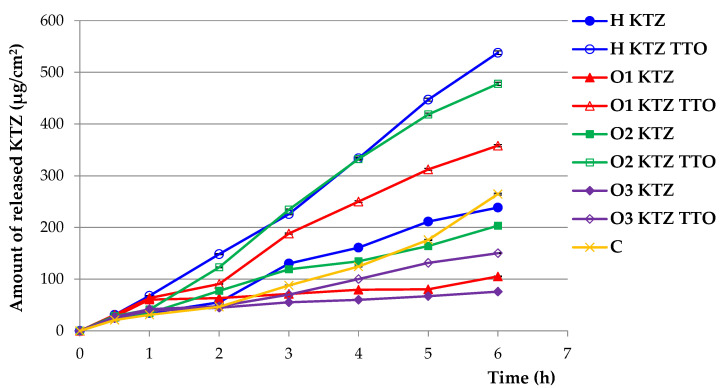
In vitro release of KTZ from prepared PLU^®^ F-127 hydrogels, PLO organogels, and commercially available product (C). Values are expressed as means ± SD of six replicates; *p* ˂ 0.05.

In the conducted study, it was shown that the amount of released KTZ from the tested formulations containing TTO was significantly higher and can be ranked in the following descending order: H KTZ TTO > O2 KTZ TTO > O1 KTZ TTO > C ≈ H KTZ > O2 KTZ > O3 KTZ TTO > O1 KTZ > O3 KTZ (Figure 4).

The largest amount of released KTZ was observed for the hydrogel containing KTZ and TTO (H KTZ TTO); after 6 h, the cumulative amount of released KTZ was 540 μg/cm^2^. The hydrophilicity of gel base facilitated the penetration of the release medium into the vehicle and promoted diffusion of the active substance. A high result (480 μg/cm^2^) was also observed for PLO with KTZ and TTO based on castor oil (O2 KTZ TTO). This might be related to the increased solubility of KTZ in the presence of TTO (Table 1). Moreover, it should be noted that PLO with castor oil containing KTZ and TTO had the lowest viscosity among the organogels containing TTO (Table 3). The obtained result shows that, in the case of this formulation, the viscosity may have a significant impact on the release of the active substance, and its value was inversely proportional to the amount of the released active substance. Similar results were obtained in the release study of etodolac and silymarin from PLOs [10,11] or croconazole and piroxicam from microemulsion-based gel formulations [47,48], where the amount of the released active substance increased with the decrease in the viscosity of the preparation. It should be noted that TTO improved the release of KTZ for all the prepared gel formulations. KTZ solubility was higher in gel bases due to the presence of essential oil and hydrophobic liquids (isopropyl myristate or oils), the components in which KTZ is more soluble than in water because it has much lower polarity. The affinity of the gel base for the active compound correlates to its solubility in the base. On the other hand, the lowest amount of released KTZ was recorded in the case of the organogels based on paraffin oil or isopropyl myristate without the addition of TTO (after 6 h, the cumulative amount of released KTZ was only 80 µg/cm^2^ and 100 µg/cm^2^, respectively). The release rate from more hydrophobic PLO formulations was slower than from hydrogel, owing to the partitioning of the KTZ between the aqueous (release medium) and oil phase (PLO).

The use of appropriate mathematical models allows the mechanism of drug release from a dosage form and the drug release kinetics to be explained. In order to better characterize the drug release behaviour, the mechanism of KTZ release from the prepared gel formulations was investigated according to four mathematical models: the zero-order kinetic, first-order kinetic, Higuchi, and Korsmeyer–Peppas models. Zero-order kinetics describes the drug release rate from dosage forms independent of the drug concentration, where the active substance is released slowly. Alternatively, the first-order model best describes a release process that is directly proportional to the drug concentration embedded in the vehicle. Higuchi’s mathematical model suggests a pure diffusion release mechanism of the active substance from a vehicle, with no erosion or swelling of the matrix. The Korsmeyer–Peppas model can be used as a decision parameter between the Higuchi and zero-order models [49,50,51]. The kinetic model describing process of KTZ release from the developed gel preparations was selected according to the highest correlation coefficient value (*R*^2^) and the values of the release exponent (*n*).

The most fitted model to explain the drug release from all the prepared formulations was the zero-order kinetic model (Table 5).

Zero-order kinetic plots were found to be fairly linear as indicated by their highest regression values, and for all the prepared formulations their correlation coefficient values (*R*^2^) were in the range of 0.870–0.995. These results are in agreement with the authors who reported the zero-order kinetic as the best fitted model to describe the release of propolis and silymarin from PLO organogels [11,13]. Moreover, zero-order kinetics has been observed for morphine-loaded and clotrimazole pluronic gels [35,49]. A prolonged release of the incorporated drug was achieved, suggesting better patient compliance and higher therapeutic efficacy. However, in the case of the commercially available product (control), the Korsmeyer–Peppas model was proved to be the leading model to describe the drug release.

Another relevant parameter to characterize the different release mechanisms of the drug is the diffusion exponent *n*. If the *n* value is ≤0.5, the release mechanism follows Fickian diffusion. When *n* = 1.0, the release takes place with zero-order kinetics and values of *n* 0.5 < *n* < 1 correspond to a non-Fickian anomalous transport model [50,51]. Exponents for KTZ diffusion from the prepared gel formulations and control formulation were in the range of 0.5 ˂ *n* < 1, suggesting anomalous transport, a combination of more than one release mechanism, governed by both abovementioned processes.

### 2.5. Antifungal Activity

Due to the increased occurrence of skin fungal infections and their growing drug resistance, new options for their effective treatment are being investigated, which can be based on monotherapy or combined therapy. Within the group of natural substances, essential oils and their constituents have attracted growing interest in recent years, being preferred over the commonly used synthetic compounds as they are regarded as safe (nontoxic, non-irritating, non-allergenic), compatible with drugs and other excipients, and suitable skin penetration promotors for both hydrophilic and lipophilic drugs. Moreover, essential oils and their constituents possess reported biological activities, including antibacterial, antiviral, and antifungal effects. Thus, the antimycotic activity of tea tree oil (TTO) against various pathogenic fungi species was recently described. It is attributed to the high level of terpinen-4-ol in the composition of TTO. TTO can be combined with common antifungal drugs, such as KTZ, to enhance its efficacy after topical administration, improve the skin penetration, and intensify the therapeutic effect [25,27].

In order to evaluate the antifungal effectiveness of the prepared formulations, their activity against *C. albicans*, *C. parapsilosis*, and *C. krusei* was tested. *Candida* strains are a commonly chosen model test organism. Studies investigating the mechanisms of antifungal action revealed that TTO alters the permeability of *Candida* cells. It was also demonstrated that the membrane fluidity of *C. albicans* cells treated with 0.25% TTO was significantly increased, confirming that the oil substantially alters the membrane properties of *C. albicans* [25,52]. Additionally, TTO inhibits respiration in *C. albicans* in a dose-dependent manner [25,53].

**Figure 5:**
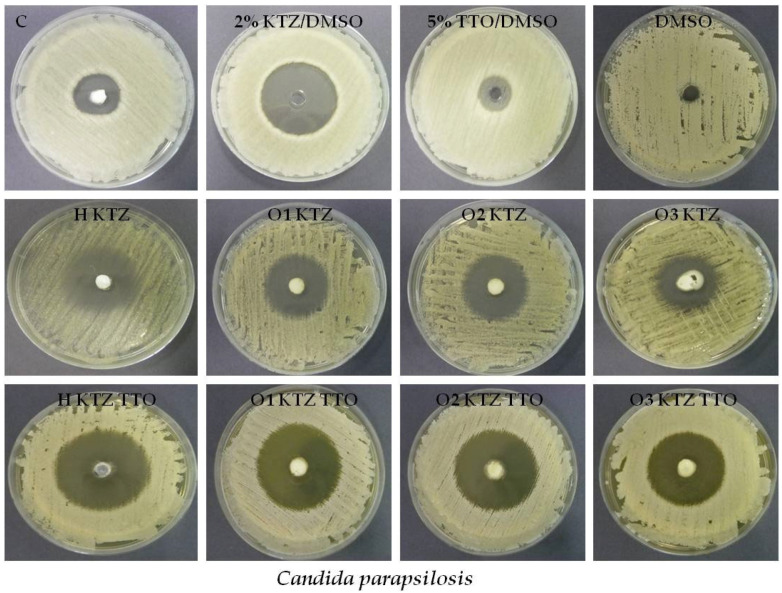
Antifungal activity against *C. parapsilosis* of prepared PLU^®^ F-127 hydrogels and PLO organogels containing KTZ (H KTZ, O1 KTZ, O2 KTZ, O3 KTZ), KTZ and TTO (H KTZ TTO, O1 KTZ TTO, O2 KTZ TTO, O3 KTZ TTO), and controls: DMSO (dimethyl sulfoxide), KTZ 2% in DMSO, TTO 5% in DMSO, and commercially available product (C).

Based on the obtained data, it was found that the addition of TTO improved the antifungal properties of all the formulations against the tested strains, and the highest inhibition zones were observed in the case of *C. parapsilosis* (Table 6, Figure 5). The highest antifungal activity against the tested *Candida* strains was observed for hydrogels. This might be due to the relatively weaker diffusion of KTZ from PLO bases with a lipophilic component. Gel preparations containing only KTZ possessed antifungal properties similar to those of the commercially available product. The placebo gels did not show any antimycotic activity. Our results are in the accordance with the study which demonstrated that combining TTO and a conventional drug such as fluconazole may help treat difficult yeast infections [27].

### 2.6. Permeation Study of KTZ

Essential oils and their constituents have been widely investigated as safe and suitable skin penetration enhancers for both hydrophilic and hydrophobic drugs. It was shown that TTO applied topically decreased the skin integrity in a dose-dependent manner [54,55]. Essential oils and their constituents, mainly terpenes, are considered as non-toxic penetration enhancers of active agents whose mode of action is based on either disintegration of intercellular lipids between corneocytes in the epithelial layer or interactions with intercellular proteins which leads to their conformational changes [56,57,58]. Despite a number of research studies devoted to the permeation behaviour of TTO through the skin barrier [54,58], little data exists on the use of TTO in promoting the penetration of drugs when applied topically. Therefore, the goal of this study was to examine the in vitro TTO potential as a promoting agent through the Strat-M membrane with a high resemblance to the human skin [59]. We assumed that the presence of TTO in KTZ-loaded gels modulates the absorption and accumulation of the drug.

The penetration model was used in this study to evaluate the passive diffusion of KTZ across the artificial skin membrane. To assure a maximal penetration rate, an infinite dose of the drug embedded in gels was applied on the membrane surface. KTZ being a weak dibasic agent (with two pKa values: 3.96 and 6.51) [60] is uncharged at a pH greater than the pKa value and thus is more likely to cross the membrane. To diminish perturbances related to the presence of KTZ in an ionized or unionized state, prior to its application on the membrane surface, each gel formulation was initially mixed with a phosphate buffer (pH 6.8).

**Figure 6:**
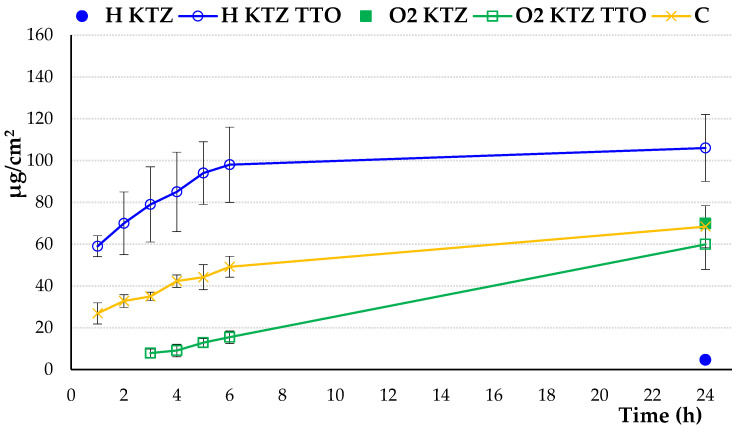
In vitro permeation profile (expressed as the amount of drug permeated to acceptor phase per unit membrane area) of ketoconazole (KTZ) from hydrogel (H) and organogel (O2) formulations with pure drug or combination of drug and tea tree oil (TTO) as compared to control (commercial topical preparation with KTZ (C)). Values are expressed as means ± SD of six replicates; *p* ˂ 0.05.

Table 7 presents data obtained from penetration studies, whereas Figure 6 shows the cumulative amount of KTZ from the designed hydrogels (H KTZ; H KTZ TTO), PLOs O2 (O2 KTZ; O2 KTZ TTO), and the commercially available preparation permeated over time through the Strat-M membrane. As in the tests using the formulations H KTZ and O2 KTZ, drug levels in the acceptor fluid were below detectable limits in early test points; in Figure 6 the cumulative amount of KTZ permeated from the designed gels after only 24 h is presented.

Profound differences in the KTZ penetration pattern among the tested formulations were found. Basically, the KTZ penetration rate from the hydrogel and organogel formulations containing TTO was greater than those obtained from preparations with pure KTZ (Table 7). A particularly visible effect of TTO was observed between the tested hydrogel formulations. Hydrogel H KTZ TTO displayed the highest amount of permeated KTZ among the tested formulations (with over 105 µg/cm^2^ of the drug present in the acceptor medium after 24 h), whereas H KTZ showed the weakest penetration behaviour (less than 5 µg/cm^2^ of KTZ in the acceptor medium after 24 h).

O2 KTZ TTO displayed almost 2-fold lower values of permeation as compared to H KTZ TTO, suggesting that KTZ has a stronger affinity for lipophilic O2 compounds than for Strat-M membrane lipids. It is worth noting that in tests with formulations without TTO, drug levels in the acceptor phase were below detectable limits over the first 6 h of studies (Table 7). In turn, a relatively high KTZ concentration was present in the acceptor phase at the initial stage of the test with hydrogel H KTZ TTO and the commercial topical KTZ preparation (Figure 6). An observed enhanced penetration was most probably a result of the increased solubility of the drug in the presence of TTO (Table 1). These results are also in agreement with the data from the dissolution study, where a profoundly faster drug release was noticed for the hydrogel formulation containing KTZ TTO (Figure 4). Despite the presence of penetration promoters, the amount of the total permeated and accumulated drug fractions in the control studies with commercial KTZ dermal preparation were profoundly lower than those obtained for the hydrogel formulation.

**Figure 7:**
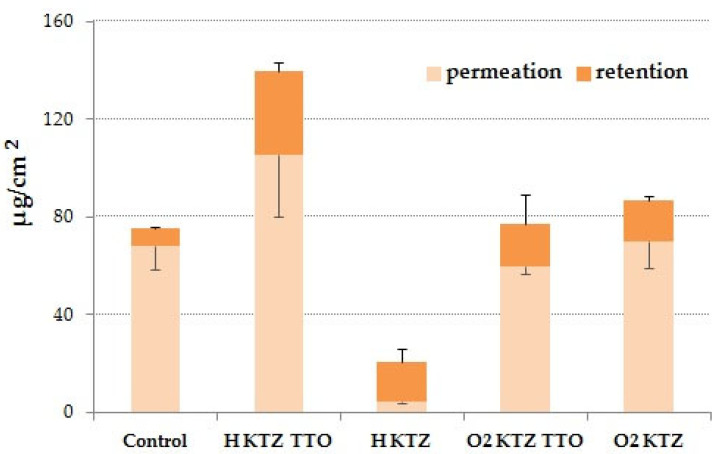
In vitro permeation and retention (expressed as total amount of agent permeated or accumulated per unit membrane area) of ketoconazole (KTZ) from hydrogel (H) and organogel (O2) formulations with pure drug or combination of drug and tea tree oil (TTO) in membrane imitating human skin attained after 24 h; C—commercial topical preparation with KTZ. Values are expressed as means ± SD of six replicates; *p* ˂ 0.05.

The amount of KTZ recovered from the membrane surface at the end of the studies (Table 7) was relatively high and confirmed the accuracy of the applied research method. From Figure 7, it can be seen that the degree of drug accumulation in tests with the designed hydrogel and organogel formulations was relatively higher compared to that of control studies. A clear impact of TTO on drug accumulation was observed in the hydrogel formulations and significantly higher retention values of KTZ from H KTZ TTO in the artificial skin were noted. Surprisingly, there were no differences in retention behaviour of KTZ from the organogel formulations. In fact, both tested organogels displayed similar values of drug accumulation in Strat-M as compared to H KTZ.

Based on the obtained results, it might be assumed that the designed hydrogel formulation with TTO enables a faster rate of KTZ permeation and simultaneously its greater retention at the application site, which makes it promising as a dermal carrier for the treatment of fungal infections.

## 3. Materials and Methods

### 3.1. Materials

Ketoconazole (KTZ) was received as a gift sample from Polfarmex S.A. (Kutno, Poland). Castor oil and liquid paraffin were purchased from Pharma Cosmetics (Kraków, Poland). Soybean lecithin (with phosphatidylcholine content min. 50% *w*/*w* (53.1% *w*/*w*) and lysophosphatidylcholine max. 6% *w*/*w* (0.6% *w*/*w*)) was from Lipoid GmbH (Ludwigshafen am Rhein, Germany). Isopropyl myristate and the Strat-M Membrane^®^ (25 mm) used during the permeation study were provided by Merck (Hohenbrunn, Germany). Tea tree oil (TTO; ≤10% cineole, ≥34% terpin-4-ol), Igepal^®^ CA-630 (octylphenoxy poly(ethyleneoxy)ethanol), DMSO, and Pluronic^®^ F-127 (amphiphilic copolymers consisting of units of ethylene oxide and polypropylene oxide; ~12,600 g/mol) were obtained from Sigma Aldrich (St. Louis, MO, USA). Propylene glycol, sodium acetate anhydrous, sodium hydroxide potassium, potassium phosphate monobasic, sodium phosphate dibasic, methanol (Chempur, Piekary Śląskie, Poland), ethanol 99.9% (J.T. Baker, Deventer, The Netherlands), acetonitrile HPLC grade, and ethyl alcohol 96% (POCH, Gliwice, Poland) were used as received. All chemicals and solvents used for the experiments were of analytical grade. Control *Candida* strains, *Candida albicans* ATCC 10231, *Candida krusei* ATCC 6528, and *Candida parapsilosis* ATCC 22,019, along with Sabouraud Dextrose LAB-AGAR™ were provided by Biomaxima (Lublin, Poland). Cuprophan^®^ was received from Medicell (London, UK). Nizoral^®^ cream (20 mg/g; McNeil Healthcare, Dublin, Ireland) with the composition of 20 mg/g of ketoconazole, propylene glycol, stearyl alcohol, cetyl alcohol, sorbitan stearate, Polysorbate 60, isopropyl myristate, sodium sulfite anhydrous, Polysorbate 80, and purified water, was used as a control. Cellulose acetate and nylon membrane filters (CA, NY 0.45 μm) were received from Millipore (Billerica, MA, USA). Hairless mice skin from CBy.Cg-Foxn1nu/J was obtained from the Experimental Medicine Center of the Medical University of Białystok (the skin was from hairless mice intended for the collection of organs and this procedure did not require the approval of the Local Ethical Committee on Animal Testing). Samples of the skin were stored at −20 °C and were defrosted before the experiment and cut into 5 mm diameter pieces.

### 3.2. Solubility Studies

The solubility of KTZ was determined by using the shake-flask method. In brief, an excess amount of KTZ was added to each vial containing 5 mL of the selected solvents (water, isopropyl myristate, castor oil, paraffin oil, and the abovementioned solvents with the addition of 5% TTO). Mixtures were mechanically shaken for 24 h at 25 °C ± 0.5 °C and allowed to stand for 24 h to attain equilibrium. Then, the samples were centrifuged at 4000 rpm for 15 min, followed by filtration through a CA membrane filter (0.45 μm), diluted appropriately with methanol, and analyzed by the HPLC method at 231 nm against a standard.

### 3.3. Preparation and Physicochemical Characteristics of PLU^®^ F-127 Gels

#### 3.3.1. Preparation of PLU^®^ F-127 Hydrogels and PLO Organogels

Different types of gel formulations were developed: PLU^®^ F-127 hydrogel (H) and pluronic/lecithin organogels (PLOs) based on isopropyl myristate (O1), castor oil (O2), and paraffin oil (O3). The gels were prepared by using a RZR 2020 mechanical stirrer (Heidolph Instruments, Schabach, Germany) according to the method described in the literature with slight modifications [10,11]. Active ingredients of KTZ at 2.0% (*w*/*w*) and TTO at 5.0% (*w*/*w*) were uniformly dispersed in previously prepared hydrogel vehicles or added to the lipid phase of the PLOs. The concentration of KTZ and TTO was set based on commercially available products and according to the literature data [22]. Control gel formulations without KTZ and TTO (placebo) were also initially prepared.

Pluronic^®^ F-127 hydrogel (H) was prepared by dispersing the polymer with a mechanical stirrer in cold water containing propylene glycol (as humectant) and potassium sorbate (as preservative). The vessel with ingredients was ice-cooled to maintain a temperature of approximately 4 °C. Stirring was continued until the gelling agent was completely dissolved and formed a clear solution.

PLO gels were prepared by mixing of the aqueous and oil phase at high shear. The aqueous phase was received by dissolving a weighed quantity of Pluronic^®^ F-127 (15.5%, *w*/*w*) in an aqueous-based solution containing propylene glycol and potassium sorbate. The oily phase was prepared by dissolving lecithin (5%, *w*/*w*) in isopropyl myristate, castor oil, or paraffin oil, respectively. After preparation, the water phase and the oil phase were conditioned at 4 °C for 24 h in order to completely dissolve the ingredients and stabilize the systems. Pluronic/lecithin organogels (PLOs) were obtained by combining water and oil phases in different weight ratios, 30:70, 50:50, and 70:30, using a mechanical stirrer at a speed of 400 rpm. A stable and homogeneous gel system was obtained at a weight ratio of water phase to oil phase of 70:30. KTZ and TTO were added to the lipid phase before organogel formation. The compositions of the designed formulations are listed in Table 2.

#### 3.3.2. Organoleptic Characteristics

Each formulation was organoleptic tested for odor, colour, texture, phase separation, and greasiness.

#### 3.3.3. KTZ Content Analysis by HPLC Method

KTZ content was determined after extraction of the gel formulation samples using ethanol 99.9% and analysis by the HPLC method with an Agilent Technologies 1200 HPLC system equipped with a G1312A binary pump, a G1316A thermostat, a G1379B degasser, and a G1315B diode array detector (Agilent, Waldbronn, Germany) in the following conditions: a Zorbax Eclipse XDB-C18, 4.6 × 150 mm, 5 µm column (Agilent, Waldbronn, Germany); mobile phase: an acetonitrile–methanol–phosphate buffer, pH 6.8 (40:35:25, *v/v*); a flow rate of 1.0 mL/min; detection at 231 nm; and a retention time of 4.0 min [61]. The standard calibration curve was linear over the range of 2.5–65 µg/mL (*R*^2^ = 0.998; y = 29.676x − 24.455).

#### 3.3.4. pH Determination

The pH was measured using the glass electrode of a Orion 3-Star pH Meter (Thermo Scientific, Waltham, MA, USA). Each measurement was carried out six times and the average pH was calculated.

#### 3.3.5. Particle Size Analysis

Gel formulations samples (in quantities corresponding to 10 μg of KTZ) were observed under 100× magnification and particle size was analyzed by using an optical microscope Motic BA 400 equipped with a camera (Moticon, Wetzlar, Germany) [62].

#### 3.3.6. Morphological Analysis of PLU^®^ F-127 Hydrogels by Scanning Electronmicroscopy (SEM)

Samples of the PLU^®^ F-127 hydrogels were dried to dehydrate and remove moisture at 40 °C for 48 h (Incubator BD Avantgarde.Line, Binder, Tuttlingen, Germany). Afterwards, the samples were sputter-coated with a thin layer of gold (6.5 nm) in an argon atmosphere (Leica EM AC 2000, Wetzlar, Germany) and imaged using SEM (Inspect™ S50, FEI Company, Hillsboro, OR, USA).

#### 3.3.7. Determination of Rheological Properties

The viscosity was determined using a Brookfield viscometer (RVDV-III Ultra, Brookfield Engineering Laboratories, Middlebro, MA, USA) equipped with a cone/plate type CPA52Z measuring system (plate diameter: 24 mm; cone angle: 3°) at a temperature of 25 °C ± 1 °C. The viscosity values of the gel formulations at shear rate 6.00 s^−1^ were recorded and the rheograms were estimated by plotting the obtained values of shear stress versus shear rate (2.00–10.00 s^−1^).

#### 3.3.8. Texture Analysis

The texture analysis was performed using a Texture Analyser TA.XT Plus (Stable Micro System, Godalming, UK) equipped with a back extrusion ring A/BE of 35 mm for backwards extrusion measurements. In the analysis, compressibility was given by the area under the positive curve, firmness from the maximum value of the positive curve, and adhesiveness from the area under the negative curve. A ring was pushed at a speed of 2 mm·s^−1^ for a distance of 5 mm into the sample (30 g) and redrawn. The textural properties of the formulations were calculated via the instrument software. Parameters such as firmness, compressibility, and adhesiveness were determined from the force-time plots [40,63].

### 3.4. Ex Vivo Bioadhesive Properties

Evaluation of bioadhesiveness was performed using a TA.XT.Plus Texture Analyser (Stable Micro Systems, Godalming, UK) on the hairless mice skin model. On the day of the experiment, the skin was defrosted and cut into 5 mm diameter pieces and immersed in a physiological saline solution (0.9% NaCl) at 25 °C ± 0.5 °C for 30 min. Next, the skin was attached to the lower end of a cylindrical probe using a cyanoacrylate glue, and gel formulations in the amount of 0.5 g were placed below the probe. The experiment was carried out at 32 °C ± 0.5 °C to mimic the in vivo conditions, according to the previously developed procedure [63]. Bioadhesiveness was determined as the maximum detachment force (F_max_) and the work of adhesion (W_ad_). The work of adhesion (W_ad_) was estimated with the use of the following formula:

W_ad_ = A × 0.1 × 1000 (1)

where A is the area under the force versus the distance curve, multiplied by 0.1—conversion time measurement to distance (the sampler was raised at 0.1 mm·s^−1^)—then multiplied by 1000 in order to express the result in units of work µJ [40,63].

### 3.5. In Vitro KTZ Release

The release of KTZ was measured through a cellulose membrane (Cuprophan^®^, Medicell, London, UK) with the use of an enhancer cell with a surface area of 3.80 cm^2^. The enhancer cell consisted of a Teflon load ring, a cap, a membrane, and a drug reservoir. This study was performed with a USP dissolution apparatus 2 (Agilent 708-DS, Agilent Technologies, Cary, NC, USA) with mini vessels (250 mL) and mini paddles. Gel samples (about 3 g), were placed in the enhancer cell which was then immersed in the dissolution vessel containing 100 mL of the release medium: acetic buffer, pH 5.5 with 1% SDS to provide the sink conditions, previously warmed to 32 °C ± 0.5 °C. Agitation was provided by mini paddles at 75 rpm and aliquots of 2 mL each were withdrawn at different time intervals (0.5, 1, 2, 3, 4, 5, and 6 h). The withdrawn samples were replaced by equal volumes of fresh release medium. The samples were assayed by the HPLC method as described above.

#### Mathematical Modeling of the KTZ Release

In order to explain the drug release mechanism, data obtained from the release study were investigated with the use of various mathematical plots [50,51].

Zero-order kinetics:

F = *k* × *t* (2)

First-order kinetics:

lnF = *k* × *t* (3)

Higuchi model:

F = *k* × *t*^1/2^ (4)

Korsmeyer–Peppas model:

F = *k* × *t*^n^ (5)

where F is the fraction of the released drug, *k* is the constant connected with release, and *t* is the time.

### 3.6. Antifungal Activity

The in vitro antifungal activity of the designed gel formulations was evaluated by the agar diffusion method. Tested microorganisms included the following *Candida* strains: *C. albicans* ATCC 10231, *C. krusei* ATCC 6528, and *C. parapsilosis* ATCC 22019. Broth cultures (100 µL), adjusted to yield approximately 10^6^ CFU/mL (density corresponding to 0.5 McFarland scale), were inoculated on plates containing Sabouraud medium. After 15 min of drying at room temperature, wells with a diameter of 5 mm were cut out in agar plates, into which the prepared gel formulations (100 mg) were placed. The plates were incubated at 37 °C for 48 h. The results were recorded by measuring the zones of growth inhibition surrounding the wells using a caliper (Mitutoyo, Kawasaki, Japan) with an accuracy of 0.1 mm. Plain gels (without KTZ or TTO), DMSO, KTZ in DMSO (2%), TTO in DMSO (5%), and the commercially available product were used as the control [64,65].

### 3.7. Permeation Study of KTZ

Based on the data from the dissolution studies, hydrogel (H) and organogel (O2) formulations with pure KTZ or combination KTZ with TTO were selected for the in vitro penetration tests through the artificial membrane Strat-M^®^, which presents a high structural resemblance to human skin [59]. Studies were carried out using am in line cell system equipped with thermostated Teflon diffusion chambers (SES GmbH Analyse system) according to the method described previously by our group [60]. Prior testing gels were mixed with a phosphate buffer (pH 6.8) in the mass ratio of 2:1. The artificial membrane (with a diffusion area of 0.81 cm^2^) was placed between the donor and acceptor compartments, wetted with acceptor medium, and then the proper amount of diluted formulations (the amount of which corresponded to a 5 mg KTZ dose) was carefully applied on the membrane surface. The donor cell-compartment remained closed with a cap throughout the studies and the system was maintained at a temperature of 32 °C. A phosphate buffer (pH 6.8) was applied as an acceptor phase and the use of 0.5% (*w*/*w*) Igepal^®^ was found to be a prerequisite to assure sink conditions. The medium was flowed beneath the artificial membrane in a close-loop system at a constant rate of 35 mL/h. Commercially available cream with KTZ registered for dermal delivery was used as a control. At determined time intervals, samples of the acceptor medium were withdrawn, filtered, and analyzed for KTZ content with HPLC. The samples were replaced by the same volume of acceptor phase.

At the end of the studies, diluted formulations from the donor compartment were collected into glass flasks and the membrane surface was carefully washed with phosphate buffer (pH 6.8) and methanol until complete KTZ removal from the donor chamber. To assess the amount of the drug retained in the artificial membrane, incubation in methanol (5 h at 30 °C with continuous shaking at 150 rpm) preceded with a sonification step (5 min at 30 °C) was applied. The filtered extract was next analyzed by the HPLC method. Penetration and retention behaviour were expressed as the amount of KTZ permeated to the acceptor medium per membrane unit area.

### 3.8. Statistical Analysis

Results are presented as the mean ± standard deviation (SD) based on six independent experiments. Statistical analysis was done by one-way analysis of variance (ANOVA) using Statistica 12.0 software (StatSoft, Kraków, Poland). A probability level of *p* < 0.05 was considered as significant.

## 4. Conclusions

The formulations intended for use on the surface of skin affected by fungal infection should be characterized by high antimycotic effectiveness, favourable application properties, and good release of the active substance from the vehicle. In the present study, ketoconazole, a poorly water-soluble antifungal drug, and TTO having confirmed antimycotic activity were successfully formulated into different types of Pluronic^®^ F-127 gel preparations (hydrogel and three PLO organogels). The results of the physicochemical characterization tests proved that all prepared formulations corresponded to the official recommendations in terms of drug content and pH. The rheological tests demonstrated that the studied gels exhibited non-Newtonian pseudoplastic behaviour with a thixotropic character, adequate viscosity, and textural properties. Moreover, all developed formulations possessed bioadhesive features. The addition of TTO essential oil increased the solubility of KTZ, improved its release and permeation rate from the vehicles through the synthetic membranes, and enhanced antifungal activity against the tested *Candida* strains (especially in the case of *C. parapsilosis*). The most promising gel vehicles for topical delivery of KTZ are hydrogel and PLOs based on castor oil with the addition of TTO. It may be concluded that the designed formulations containing KTZ with TTO could be a valuable alternative for the treatment of skin fungal infections.

## Figures and Tables

**Table 1:** Solubility of KTZ in chosen solvents. Values are expressed as means ± SD of six replicates.

Tested Solvent	Solubility of KTZ (μg/mL)
Water	9.56 ± 0.09
Isopropyl myristate	93.99 ± 3.26
Castor oil	93.55 ± 8.39
Paraffin oil	17.02 ± 2.84
Water + TTO 5%	35.70 ± 4.59
Isopropyl myristate + TTO 5%	130.95 ± 5.13
Castor oil + TTO 5%	154.78 ± 7.94
Paraffin oil + TTO 5%	80.11 ± 6.5

**Table 2:** Compositions of designed gel formulations (PLU^®^ F-127 hydrogels and PLO organogels).

Ingredient (g)	Formulation Code											
	Placebo Gels				KTZ Gels				KTZ and TTO Gels			
	H	O1	O2	O3	H KTZ	O1 KTZ	O2 KTZ	O3 KTZ	H KTZ TTO	O1 KTZ TTO	O2 KTZ TTO	O3 KTZ TTO
Ketoconazole (KTZ)	-	-	-	-	2	2	2	2	2	2	2	2
Tea tree oil (TTO)	-	-	-	-	-	-	-	-	5	5	5	5
Pluronic^®^ F-127	15.5	15.5	15.5	15.5	15.5	15.5	15.5	15.5	15.5	15.5	15.5	15.5
Potassium sorbate	0.2	0.2	0.2	0.2	0.2	0.2	0.2	0.2	0.2	0.2	0.2	0.2
Propylene glycol	10	10	10	10	10	10	10	10	10	10	10	10
Purified water	up to 100	up to 100	up to 100	up to 100	up to 100	up to 100	up to 100	up to 100	up to 100	up to 100	up to 100	up to 100
Lecithin	-	5	5	5	-	5	5	5	-	5	5	5
Isopropyl myristate	-	up to 100	-	-	-	up to 100	-	-	-	up to 100	-	-
Castor oil	-	-	up to 100	-	-	-	up to 100	-	-	-	up to 100	-
Paraffin oil	-	-	-	up to 100	-	-	-	up to 100	-	-	-	up to 100

**Table 3:** Drug content, pH, particle size, and viscosity of prepared PLU^®^ F-127 hydrogels and PLO organogels containing KTZ (H KTZ, O1 KTZ, O2 KTZ, O3 KTZ), KTZ and TTO (H KTZ TTO, O1 KTZ TTO, O2 KTZ TTO, O3 KTZ TTO), placebo gels (H, O1–O3), and commercially available product (C). Values are expressed as means ± SD of six replicates.

Formulation Code	Drug Content (%)	pH	Particles Size (µm)	Viscosity * (mPa∙s)
C	97.5 ± 1.5	6.40 ± 0.02	37 ± 14	5920 ± 69
H	-	6.80 ± 0.01	-	2128 ± 40
H KTZ	92.1 ± 0.2	6.80 ± 0.01	63 ± 42	3142 ± 85
H KTZ TTO	90.4 ± 1.1	6.60 ± 0.02	38 ± 27	23,548 ± 153
O1	-	6.70 ± 0.01	-	19,861 ± 127
O1 KTZ	96.4 ± 1.2	6.80 ± 0.01	62 ± 41	21,652 ± 78
O1 KTZ TTO	96.8 ± 1.3	6.60 ± 0.01	36 ± 24	23,399 ± 176
O2	-	6.70 ± 0.01	-	6041 ± 82
O2 KTZ	95.8 ± 1.0	6.70 ± 0.01	58 ± 38	8498 ± 105
O2 KTZ TTO	96.3 ± 1.8	6.60 ± 0.01	48 ± 26	18,995 ± 96
O3	-	6.60 ± 0.01	-	11,829 ± 74
O3 KTZ	91.0 ± 0.7	6.60 ± 0.01	63 ± 39	16,250 ± 124
O3 KTZ TTO	95.8 ± 1.7	6.50 ± 0.01	55 ± 26	21,255 ± 160

* Viscosity was measured at the shear rate 6.00 s^−1^ at 25 °C.

**Table 4:** Textural properties of prepared PLU^®^ F-127 hydrogels and PLO organogels containing KTZ (H KTZ, O1 KTZ, O2 KTZ, O3 KTZ), KTZ and TTO (H KTZ TTO, O1 KTZ TTO, O2 KTZ TTO, O3 KTZ TTO), placebo gels (H, O1–O3), and commercially available product (C). Values are expressed as means ± SD of six replicates.

Formulation Code	Firmness (g)	Compressibility (g·s)	Adhesiveness (g·s)
C	174.6 ± 0.6	253.0 ± 40.4	−346.4 ± 34.6
H	133.1 ± 47.6	143.8 ± 58.1	−186.3 ± 89.0
H KTZ	141.5 ± 32.2	184.4 ± 38.1	−250.9 ± 9.3
H KTZ TTO	369.8 ± 71.7	303.6 ± 32.9	−298.5 ± 37.8
O1	144.0 ± 77.7	194.1 ± 47.6	−274.8 ± 62.6
O1 KTZ	193.0 ± 3.5	282.9 ± 28.7	−285.7 ± 1.4
O1 KTZ TTO	258.1 ± 25.5	470.2 ± 50.1	−491.3 ± 5.4
O2	15.1 ± 2.8	35.8 ± 5.4	−62.9 ± 7.4
O2 KTZ	18.7 ± 2.5	43.8 ± 6.0	−63.6 ± 1.4
O2 KTZ TTO	212.8 ± 43.4	413.2 ± 9.8	−387.9 ± 36.5
O3	16.8 ± 4.2	39.5 ± 8.0	−64.1 ± 11.7
O3 KTZ	21.9 ± 11.6	51.4 ± 5.4	−59.2 ± 6.9
O3 KTZ TTO	333.4 ± 2.7	469.7 ± 72.3	−439.1 ± 9.5

**Table 5:** Models of KTZ release from designed formulations and commercially available product (C).

Formulation Code	Kinetic Models								
	Zero-Order Kinetics		First-Order Kinetics		Higuchi Model		Korsmeyer–Peppas Model		
	*R* ^2^	K_0_	*R* ^2^	K_I_	*R* ^2^	K_H_	*R* ^2^	K_KP_	*n*
C	0.987	0.266	0.937	0.197	0.853	0.808	0.992	0.712	0.631
H KTZ	0.980	0.262	0.942	0.180	0.942	0.819	0.971	0.638	0.584
H KTZ TTO	0.995	0.591	0.915	0.211	0.953	1.843	0.979	0.694	0.716
O1 KTZ	0.870	0.070	0.718	0.078	0.846	0.225	0.796	0.194	0.562
O1 KTZ TTO	0.989	0.392	0.921	0.186	0.959	1.233	0.975	0.627	0.609
O2 KTZ	0.988	0.203	0.902	0.160	0.978	0.645	0.968	0.570	0.529
O2 KTZ TTO	0.992	0.555	0.907	0.228	0.963	1.745	0.971	0.797	0.753
O3 KTZ	0.980	0.054	0.853	0.077	0.952	0.173	0.927	0.230	0.826
O3 KTZ TTO	0.977	0.144	0.968	0.133	0.921	0.446	0.976	0.445	0.524

**Table 6:** Antifungal activity of prepared PLU^®^ F-127 hydrogels and PLO organogels containing KTZ (H KTZ, O1 KTZ, O2 KTZ, O3 KTZ), KTZ and TTO (H KTZ TTO, O1 KTZ TTO, O2 KTZ TTO, O3 KTZ TTO),and controls: placebo gels (H, O1–O3), KTZ 2% in DMSO, TTO 5% in DMSO, and commercially available product (C). Values are expressed as means ± SD of six replicates.

Formulation Code	Name of the Strain		
	*C. albicans* ATCC 10231	*C. parapsilosis* ATCC 22019	*C. krusei* ATCC 6528
	Zone of Inhibition (mm)		
C	28.3 ± 0.6	39.5 ± 0.8	27.0 ± 1.1
DMSO	0.0 ± 0.0	0.0 ± 0.0	0.0 ± 0.0
TTO 5%/DMSO	25.3 ± 0.5	26.0 ± 1.5	21.8 ± 1.9
KTZ 2%/DMSO	26.0 ± 0.9	50.8 ± 1.0	39.8 ± 0.4
H	0.0 ± 0.0	0.0 ± 0.0	0.0 ± 0.0
H KTZ	36.8 ± 1.9	37.2 ± 1.7	28.5 ± 1.9
H KTZ TTO	42.2 ± 0.8	50.5 ± 0.8	30.5 ± 1.4
O1	0.0 ± 0.0	0.0 ± 0.0	0.0 ± 0.0
O1 KTZ	35.2 ± 1.0	35.5 ± 0.5	28.0 ± 1.7
O1 KTZ TTO	39.7 ± 1.6	46.3 ± 1.0	28.5 ± 1.0
O2	0.0 ± 0.0	0.0 ± 0.0	0.0 ± 0.0
O2 KTZ	34.0 ± 0.6	35.0 ± 0.6	27.0 ± 0.8
O2 KTZ TTO	38.5 ± 0.8	45.7 ± 0.8	27.5 ± 0.5
O3	0.0 ± 0.0	0.0 ± 0.0	0.0 ± 0.0
O3 KTZ	26.3 ± 1.4	32.3 ± 1.4	25.7 ± 1.3
O3 KTZ TTO	37.3 ± 0.8	42.3 ± 1.3	26.8 ± 1.0

**Table 7:** Quantitative analysis of KTZ from hydrogels and organogels with KTZ or combination of KTZ and TTO as compared to control (commercially available topical product with KTZ) in acceptor medium, retained in the Strat-M and on the membrane surface (drug recovery from donor compartment) after 24 h incubation.

Compartment	Parameter	Control		H KTZ		H KTZ TTO		O2 KTZ		O2 KTZ TTO	
Acceptor medium	Total amount of permeated drug per Strat-M area (μg/cm^2^)	after 6 h	after 24 h	after 6 h	after 24 h	after 6 h	after 24 h	after 6 h	after 24 h	after 6 h	after 24 h
		49.2	68.4	n.d.	4.6	98.2	106.1	n.d.	69.9	15.5	59.9
Tissue retention ^a^	Total amount of drug per Strat-M area (μg/cm^2^) *	7.3		16.4		34.0		17.5		17.3	
Drug recovery from donor compartment ^b^	Percent of drug dose (%)	94.0		98.0		77.9		99.0		91.1	

* Formulations with KTZ or commercially available topical product with KZT (amount corresponded to a dose 5 of mg) were placed on the tissue surface with an area of 0.81 cm^2^; ^a,b^ Concentrations of KTZ were assessed in (a) solvent used for membrane extraction and (b) diluted KTZ formulations aspirated from the donor compartment; n.d.—not detected: in tests using formulations of H KTZ and O2 KTZ, drug levels in the acceptor fluid were below detectable limits in early test points (1, 2, 3, 4, 5, and 6 h).

## Data Availability

Data are contained within the article; raw data are available upon request.
